# Surgical outcomes in elective sigmoid resection for diverticulitis stratified according to indication: a propensity-score matched cohort study with 903 patients

**DOI:** 10.1007/s00423-023-03034-9

**Published:** 2023-08-03

**Authors:** Fabio Nocera, Fabian Haak, Alberto Posabella, Fiorenzo Valente Angehrn, Ralph Peterli, Beat P. Müller-Stich, Daniel C. Steinemann

**Affiliations:** 1 Clarunis, Department of Visceral Surgery, University Center for Gastrointestinal and Liver Diseases, St. Clara Hospital and University Hospital Basel, Kleinriehenstrasse 30, 4058 Basel, Switzerland; 2 Departmen of Surgery, University Hospital Basel, Spitalstrasse 23, 4031 Basel, Switzerland

**Keywords:** Diverticulitis, Elective surgery, Surgical outcomes, Classification

## Abstract

**Objective:**

Weighing the perioperative risk of elective sigmoidectomy is done regardless of the specific diverticulitis classification. The aim of this study is to evaluate surgical outcomes according to the classification grade and the indication.

**Methods:**

All patients who underwent elective colonic resection for diverticulitis during the ten-year study period were included. They were divided into two groups: relative surgery indication (RSI) and absolute surgery indication (ASI). RSI included microabscess and recurrent uncomplicated disease. ASI included macroabscess and recurrent complicated disease. Propensity score-matching (PSM, 1:1) was performed.

**Results:**

585 patients fulfilled criteria for RSI and 318 patients fulfilled criteria for ASI. In the univariate analysis, RSI patients were younger (62 vs. 67.7 years, *p* < 0.001), had a higher physical status (ASA score 1 or 2 in 80.7% vs. 60.8%, *p* < 0.001), were less immunosuppressed (3.4% vs. 6.9%, *p* = 0.021) and suffered less often from coronary heart disease (3.8% vs. 7.2%, *p* = 0.025). After PSM, 318 RSI vs. 318 ASI patients were selected; baseline characteristics results were comparable. The proportion of planned laparoscopic resection was 93% in RSI versus 75% in ASI (*p* < 0.001), and the conversion rate to open surgery for laparoscopic resection was 5.0% and 13.8% in RSI versus ASI, respectively (*p* < 0.001). Major morbidity (Clavien/Dindo ≥ IIIb) occurred less frequently in RSI (3.77% vs. 10%, *p* = 0.003). A defunctioning stoma was formed in 0.9% and 11.0% in RSI vs ASI, respectively (*p* < 0.001).

**Conclusion:**

The lower risk for postoperative morbidity, the higher chance for a laparoscopic resection and the decreased rate of stoma formation are attributed to patients with recurrent uncomplicated diverticulitis or diverticulitis including a microabscess as compared to patients with complicated diverticulitis or diverticulitis and a macroabscess, and this applies even after PSM.

## Introduction

In the past, the indication for elective sigmoidectomy after complicated or recurrent diverticulitis was justified by the expected risk of free perforation during recurrence [1, 2]. Based on more recent studies however, it is known today that the risk of perforation is highest at the first episode (5–25%) and decreases with the number of further attacks to less than 1% by the 5^th^ episode [3–5]. In addition, it has been found that recurrent episodes of diverticulitis neither lead to a higher complication rate nor to a higher failure rate of conservative therapy [6]. Based on these findings, the understanding of this disease has changed and the previously propagated recommendation for resection after the second inflammatory episode must therefore be considered obsolete [7–9]. Today, the indication for elective sigmoidectomy in diverticulitis must respect individual patient-related factors such as comorbidities and the impact on quality of life. This is highlighted in particular by two recent published studies: The multicenter DIRECT trial such as the LASER trial demonstrated both in a randomized setting better results in quality of life comparing laparoscopic elective sigmoid resection with conservative treatment in patients with recurrent and persisting abdominal complaints. Both support surgery despite inherent risk of complications of operative management [10, 11].

Clinical guidelines for diverticulitis aim to use diagnostic methods and therapeutic strategies in a reasonable way, avoiding under- or overtreatment. Common across all guidelines is the assessment of the risk versus the benefit of the surgery and accordingly either recommend an operation or not [9, 12, 13]. However, none of these guidelines stratify the actual risk of surgery according to the classification of the diverticular disease or the severity of the disease. In addition, which specific groups of patients would benefit from elective surgery or at what time these patients should be operated on are questions that continue to be debated due to a lack of evidence-based data [14]. This may lead to different or misleading decision-making for elective surgery in the individual situation.

For these reasons, the aim of this study is to evaluate the surgical outcomes according to the different severities of indications, which considers the risk of the intervention, i.e., the morbidity of the surgery and the severity stadium of the disease. The evaluation of these different severities of indications enables us to stratify the specific risk of each classification grade and/or indication severity and helps us to achieve a more patient-related assessment, allowing us to perform better benefit-risk counseling.

## Methods

### Patients

All patients who underwent elective sigmoid resection for diverticulitis, as defined according to clinical and radiological diagnosis, between January 2011 and March 2020 at the University Center for Gastrointestinal and Liver Disease Clarunis (Basel, Switzerland) were included in this study.

**Fig. 1:**
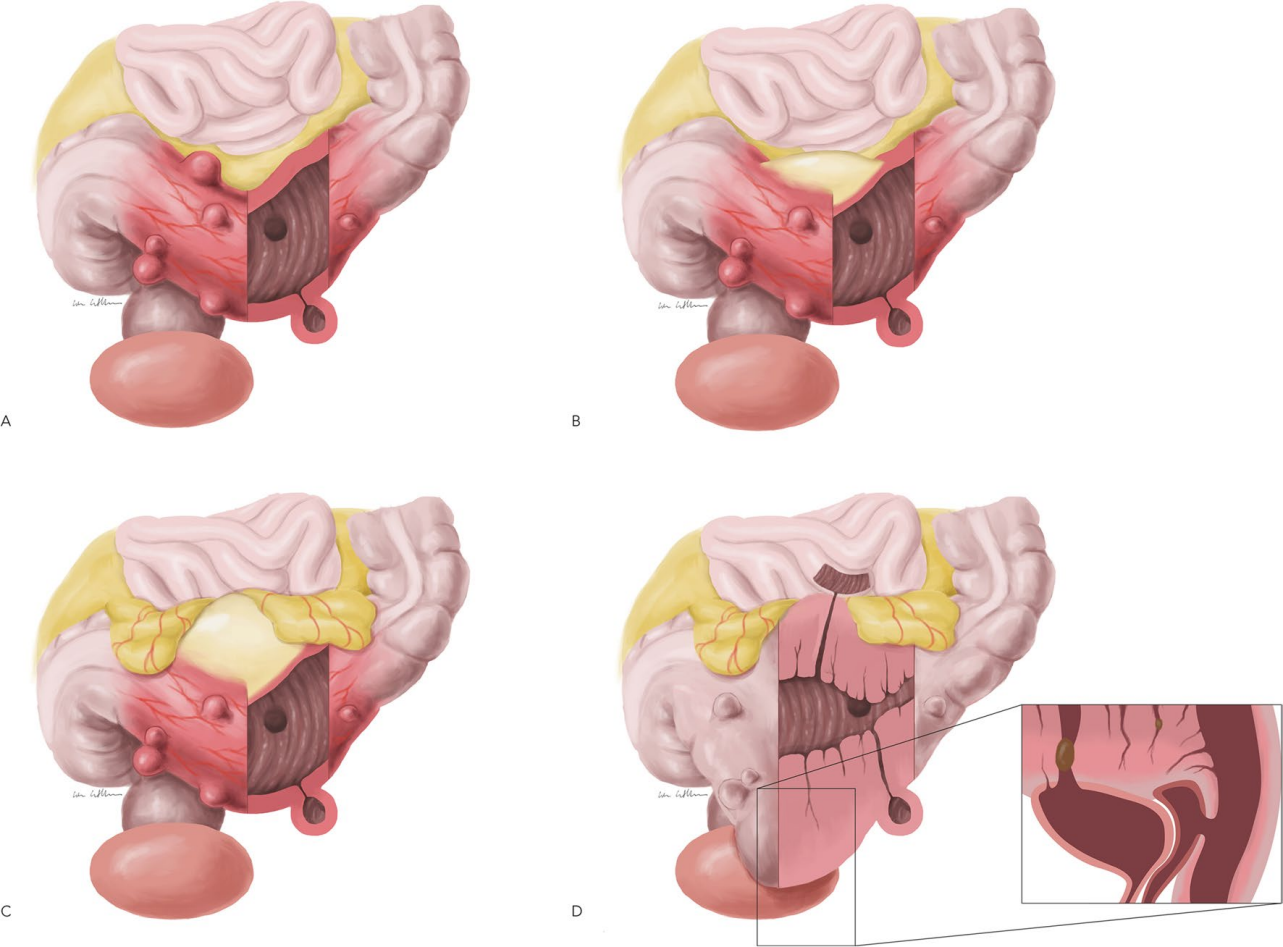
Schematic representation of diverticulitis types evaluated in this study based on CDD. **A** Acute uncomplicated sigmoid diverticulitis, **B** Sigmoid diverticulitis with microabscess, **C** Sigmoid diverticulitis with macroabscess, **D** Complicated sigmoid diverticulitis with stenosis and fistulas

The patients were divided into two groups based on the severity of the surgical indication. The severity has been evaluated by the German guidelines and the German classification system CDD (classification of diverticular disease) (Table 1) [9]. Elective sigmoidectomy has a strong indication in patients with stenosis or fistula (CDD Type IIIc) and should be offered in patients with conservatively/interventional treated macroabscess (CDD Type IIb) (Fig. 1). The less strict indication in contrast, considers patients with “milder” complaints, such as patients with recurrent uncomplicated diverticulitis (CDD Type IIIb) and patients with conservatively treated microabscess (CDD Type IIa) (Fig. 1). Due to these indications, patients were divided into a relative surgery indication group (RSI) and an absolute surgery indication group (ASI). RSI included recurrent uncomplicated diverticulitis (CDD IIIb) and surgery after complicated acute diverticulitis with a microabscess (CDD IIa). ASI included surgery after complicated acute diverticulitis with a macroabscess (CDD IIb) and patients with a complicated chronic diverticulitis with stenosis or fistula (CDD IIIc).

**Table 1:** Classification of diverticular disease (CDD) according to the German S2k guideline [9]

Term	Synonym	Definition	CDD Type
Asymptomatic diverticulosis		Identification of diverticula in colon^1^	0
Acute uncomplicated diverticulitis	Diverticulitis without perforation*^2^	Diverticulitis with phlegmonous	Ia
		Diverticulitis with phlegmonous peridiverticulitis	Ib
Acute complicated diverticulitis	Diverticulitis with covered perforation	Microabscess (< 1 cm), minimal free paracolic air	IIa
		Macroabscess (> 1 cm)	IIb
	Free perforated diverticulitis	Purulent peritonitis	IIc
		Fecal peritonitis	IIc
Chronic diverticular disease	Symptomatic uncomplicated diverticular disease (SUDD)	Typical clinical features*^3^	IIIa
	Relapsing diverticulitis without complications	Recurrent signs of inflammation	IIIb
	Relapsing diverticulitis with complications	Identifications of stenoses, fistulas, conglomerate tumor	IIIc
Diverticular bleeding		Identification of source of bleeding	IV

^*^1 = Typically diagnosed based on an incidental finding, e.g., during screening colonoscopy, *2 = Defined as a diverticulitis with or without phlegmonous peridiverticulitis, but without clinical and/or radiographic signs of free or covered perforation, *3 = This type of diverticular disease is defined by chronic relapsing or persistent symptomatic diverticular disease, which cannot be identified by means of laboratory testing, radiography or endoscopy

Exclusion criteria were emergency surgeries. The Ethical Committee of Northwestern Switzerland approved the study, and all eligible patients provided general informed consent (EKNZ Nr. 2018–00318) (Table 2).

**Table 2:** Recommendation regarding elective sigmoidectomy by different national and international guidelines [9, 12, 13, 15–18]

Indication for sigmoidectomy	DGVS/DGAV 2021 [19]	WSES 2020 [20]	WSES 2016/2013 [21, 22]	ESCP 2020 [23]	SICCR 2015 [24]	NSS 2013 [25]
Smoldering diverticulitis	*	-	-	-	-	-
Acute diverticulitis with pericolic and/or distant gas	-	○	○	○	-	
After treated macroabscess	*	-	●^1^	○	○	*
In case of therapy failure	●	●	●	●	●	-
Elective						
Related to patient-related factors	●	●	●	●	●	●
Symptomatic uncomplicated diverticulosis	○	-	-	-	○	-
Chronic uncomplicated diverticulitis	*	-	-	*^2^	*^2^	*^2^
Presence of fistula or stenosis	●	-	-	●	●	-
Prophylactic in immunosuppressed patients	○	*	*	○	*	○

● = recommended, ○ = not recommended, * = to consider,—= not discussed, 1 = pelvic abscess treated by means of percutaneous drainage due to the poor long-term outcomes of conservative treatment, 2 = in specific clinical situations (persisting symptoms and signs, degree of diverticulitis)

*DGVS/DGAV* German Society for Gastroenterology, Digestive and Metabolic Diseases/German Society for General and Visceral Surgery, *WSES* World Society of Emergency Surgery, *ESCP* European Society of Coloproctology, *SICCR* Italian Society of Colorectal Surgery, *NSS* Netherlands Society of Surgeons

### Data collection

Data from enrolled patients were included prospectively in an institutional study register database. Records were analysed retrospectively for demographic data such as age, gender, body mass index (BMI), American Society of Anaesthesiology (ASA) classification, previous intraabdominal surgeries, classification of diverticular disease (CCD), recurrent diverticulitis, number of recurrences, preoperative colonoscopy, preoperative symptoms and co-morbidities (arterial hypertension, coronary artery disease, immunosuppression and diabetes mellitus), surgical data such as surgery time (skin incision to wound closure), intraoperative complications, conversion rate to open surgery, type of access, type of anastomosis and specimen extraction technique, postoperative data such as overall morbidity according to Clavien-Dindo [26], minor morbidity (Clavien-Dindo < IIIb), major morbidity (Clavien-Dindo ≥IIIb), anastomotic leaks, mortality, comprehensive complications index (CCI) [19], number of defunctioning stomas (Hartmann procedure, planned and unplanned) and number of stoma reversals. Complications were defined as any type of deviation during surgical procedure or postoperative course. In order not to miss a recording of any complication, the recording of the complications was carried out in duplicate form in which the resident physician records the complication, and the head surgeon supervises and confirms or corrects the recording. Anastomotic leaks were diagnosed by computer tomography and/or endoscopy in cases of clinical suspicion, depending on the surgeon’s preference. A patient could suffer from more than one complication, and thus, their total number could be higher than the number of patients with complications.

### Statistics analysis

Continuous data were summarized by using mean and standard deviation (SD) for normally distributed data, or medians and interquartile range for non-normally distributed data. Student’s t-test or Mann-Witney were used to compare differences, as appropriate. Categorical variables were summarized using counts and/or percentages and were analysed with Fisher’s exact test or Pearson’s Chi-squared test. A p-value < 0.05 was considered statistically significant.

To control for potential confounders, a propensity score was generated for each patient from a multivariable logistic regression model based on key co-variables (age, BMI, arterial hypertension, coronary heart disease, immunosuppression and diabetes) as independent variables with relative or absolute indication for elective surgery as a binary dependent variable. Those baseline variables in the univariate analysis with a p value < 0.1 or otherwise of specific clinical interest were added to the model. Each patient with relative indication was matched with replacement to one patient with absolute indication in a 1:1 ratio, with a caliper width of 0.20 standard deviation of the logit of the propensity score using R statistical software version 2.13.1 using package “MatchIt” [23]. The quality of the match was assessed by comparing the patient characteristics before and after matching as shown in Table 3. Data in the matched data set were analysed using a generalised random block design for continuous variables and conditional logistic regression for categorical variables.

**Table 3:** Demographic data of patients in the RSI and ASI group

	Unmatched (*n* = 903)			Propensity score matching (PSM) (*n* = 636)		
	RSI-G *n* = 585	ASI-G *n* = 318	*p*-value	RSI-G *n* = 318	ASI-G *n* = 318	*p*-value
Age in years (median, IQR)	62 (36–92)	67.7 (26–87)	< 0.001	67.6 (43–86)	67.7 (26–87)	0.892
Gender (*N*, %)						
Male	233 (39%)	122 (38%)	0.721	108 (34%)	122 (38%)	0.283
Female	352 (61%)	196 (62%)		210 (66%)	196 (62%)	
Body mass index kg/m^2^ (median, IQR)	26.6 (14.8–45.4)	26.6 (16–48.3)	0.768	26.4 (14.8 -45.4)	26.6 (16–48.3)	0.628
ASA-Score (*N*, %)						
1–2	115 (80%)	63 (61%)	< 0.001	52 (83%)	63 (61%)	0.1
3–4	28 (20%)	40 (39%)		18 (17%)	40 (39%)	
Previous intra-abdominal surgery (*N* patients, % of patients)	316 (54%)	169 (53%)	0.834	181 (57%)	169 (53%)	0.381
Classification of diverticular disease (CDD) (*N*, %)						
IIa	80 (14%)	0		38 (12%)	0	
IIb	0	79 (25%)		0	79 (25%)	
IIIb	505 (86%)	0		280 (88%)	0	
IIIc	0	239 (75%)		0	239 (75%)	
Recurrent diverticulitis (*N* patients, %)	544 (93%)	224 (70%)	< 0.001	298 (93%)	224 (70%)	< 0.001
Number of recurrences (*N*, %)			< 0.001			< 0.001
1	35 (8%)	40 (37%)	< 0.001	16 (7%)	40 (37%)	< 0.001
2–3	188 (45%)	50 (45%)	0.915	99 (45%)	50 (45%)	1.0
4–6	181 (43%)	20 (18%)	< 0.001	98 (44%)	20 (18%)	< 0.001
> 6	16 (4%)	0	0.059	8 (4%)	0	0.056
Preoperative coloscopy (*N*, %)	390 (67%)	206 (65%)	0.609	212 (67%)	206 (65%)	0.676
Normal	339 (86%)	42 (20%)	< 0.001	182 (86%)	42 (20%)	< 0.001
Inflammation	13 (4%)	10 (5%)	0.376	7 (3%)	10 (5%)	0.466
Chronic-sclerosing	23 (6%)	33 (16%)	< 0.001	14 (7%)	33 (16%)	0.006
Fixed loop/Kinking	15 (4%)	21 (10%)	0.004	9 (4%)	21 (10%)	0.038
Stenosis	0	97 (47%)	< 0.001	0	97 (47%)	< 0.001
Fistula	0	3 (2%)	0.043	0	3 (2%)	0.119
Preoperative symptoms (*N*, %)						
Pain	273 (46%)	160 (50%)	0.297	153 (48%)	160 (50%)	0.634
Fever	20 (3%)	12 (4%)	0.851	11 (3%)	12 (4%)	1
Constipation	46 (8%)	61 (19%)	< 0.001	25 (8%)	61 (19%)	< 0.001
Diarrhea	28 (5%)	36 (11%)	< 0.001	12 (4%)	36 (11%)	< 0.001
Stool irregularities	46 (8%)	51 (16%)	< 0.001	26 (8%)	51 (16%)	0.003
Haematochezia	4 (1%)	5 (1.5%)	0.292	3 (1%)	5 (1.5%)	0.725
Nausea	11 (2%)	31 (10%)	< 0.001	7 (2%)	31 (10%)	< 0.001
Urogynaecology symptoms	4 (1%)	26 (8%)	< 0.001	1 (0%)	26 (8%)	< 0.001
General condition deterioration	15 (2%)	19 (6%)	0.016	10 (3%)	19 (6%)	0.127
Co-morbidities (*N*, %)						
Arterial Hypertension	185 (32%)	115 (36%)	0.183	114 (36%)	115 (36%)	1
Coronary heart disease	22 (4%)	23 (7%)	0.025	19 (6%)	23 (7%)	0.632
Immunosuppression	20 (3%)	22 (7%)	0.021	18 (6%)	22 (7%)	0.625
Diabetes	27 (5%)	25 (8%)	0.052	17 (5%)	25 (8%)	0.264

## Results

**Fig. 2:**
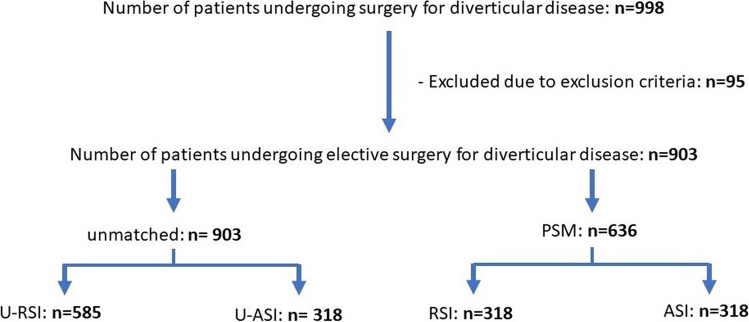
Schematic representation of patient allocation and assignment. PSM = propensity score matching, RSI = relative surgery indication, ASI = absolute surgery indication, U = unmatched

In the study period, a total of 998 operations for diverticular disease were performed at the University Center for Gastrointestinal and Liver Disease Clarunis (Basel, Switzerland). 95 patients were excluded due to exclusion criteria. The remaining 903 patients were analysed in context of this study. In the univariate analysis, 585 patients were assigned to the RSI group and 318 patients to the ASI group. After propensity score-matching (PSM) was applied, 636 remained for analysis, 318 patients for each group (Fig. 2).

### Patient characteristics

In the analysis of the unmatched cohorts, RSI patients were younger (62 {IQR 36–92} years vs. 67.7 {IQR 26–87} years, *p* < 0.001), had a better physical status (ASA score 1 or 2 in 80% vs. 61%, *p* < 0.001), were less often immunosuppressed (3.4% vs. 7.2%, *p* = 0.021 and suffered less frequently from coronary heart disease (3.8% vs. 7.2%, *p* = 0.025). BMI (*p* = 0.768), previous intraabdominal operation (*p* = 0.834) and the number of patients with arterial hypertension (*p* = 0.183) and diabetes (*p* = 0.052) were comparable. Out of the 585 patients in the unmatched RSI group, 80 (14%) patients had a Type IIa and 505 (86%) had a Type IIIb CDD (Table 3). In the unmatched ASI group, out of 318 patients, 79 (25%) patients had a Type IIb and 239 (75%) had a Type IIIc CDD. Patients in the unmatched RSI group experience more relapses prior to surgery compared to the patients in the unmatched ASI group (*p* < 0.001). Demographic data and symptoms of patients are depicted in Table 3.

After propensity score matching (PSM) controlling for age, BMI, arterial hypertension, coronary heart disease, immunosuppression, and diabetes, 318 RSI patients vs. 318 ASI patients were selected and baseline characteristics were comparable (Table 3). In RSI, 38 (12%) patients had a microabscess (Type IIa) and 280 (88%) had recurrent chronic uncomplicated diverticulitis (Type IIIb), while in the ASI group, 79 (25%) had a macroabscess (Type IIb) and 239 (75%) had a chronic complicated diverticulitis (Type IIIc). The number of previous intra-abdominal surgeries remained comparable after PSM, and in addition, the matching did not change the presence and symptomatology of the patients. Patients’ demographics are all reported in Table 3.

### Perioperative details

Perioperative details of univariate analysis are shown in the Table 4. After PSM, surgery duration was shorter in the RSI group (211 min. {IQR 60–420} vs. 229 min. {IQR 80–554}, *p* = 0.003) and the conversion rate was lower (5.0% vs. 13.8%, *p* < 0.001). Intraoperative complications were comparable between the two groups (3.1% vs. 5.3%, *p* = 0.238). However, intraoperative bleedings were less prevalent in RSI (2 {0.63%} vs. 10 {3.14%}, *p* = 0.04).

**Table 4:** Perioperative details of patients in RSI and the ASI group

	Unmatched (*n* = 903)			Propensity score matching (PSM) (*n* = 636)		
	RSI-G *n* = 585	ASI-G *n* = 318	*p*-value	RSI-G *n* = 318	ASI-G *n* = 318	*p*-value
Surgery time (min, median, IQR)	214 (60–438)	229 (80–554)	0.003	211 (60–420)	229 (80–554)	0.003
Intraoperative complications (*N*, %)	17 (2.9%)	17 (5.3%)	0.7	10 (3.1%)	17 (5.3%)	0.238
Intestinal lesion	5 (0.85%)	3 (0.94%)	1	4 (1.26%)	3 (0.94%)	1
Splenic lesion	2 (0.34%)	1 (0.31%)	1	2 (0.63%)	1 (0.31%)	1
Nerval lesion	0	1 (0.31%)	0.352	0	1 (0.31%)	1
Diaphragm lesion	1 (0.17%)	0	1	0	0	1
Bleeding	6 (1.02%)	10 (3.14%)	0.032	2 (0.63%)	10 (3.14%)	0.037
Anaesthetic problems	0	1 (0.31%)	0.352	0	1 (0.31%)	0.352
Technical stapler errors	3 (0.51%)	1 (0.31%)	1	2 (0.63%)	1 (0.31%)	1
Planned surgical access (*N*, %)			< 0.001			< 0.001
Open	24 (4%)	80 (25%)		20 (7%)	80 (25%)	
Laparoscopic	474 (81%)	209 (66%)		246 (77%)	209 (66%)	
Robot-assisted	87 (15%)	29 (9%)		52 (16%)	29 (9%)	
Conversion (*N*, %)	25 (4.3%)	44 (13.8%)	< 0.001	16 (5.0%)	44 (13.8%)	< 0.001
Surgical access including conversion (*N*, %)			< 0.001			< 0.001
Open	49 (8%)	124 (39%)	35 (11%)	124 (39%)		
Laparoscopic	450 (77%)	167 (53%)	231 (73%)	167 (53%)		
Robot-assisted	86 (15%)	27 (8%)	51 (16%)	27 (8%)		
Type of anastomosis			< 0.001			0.114
Hand-sewen	9 (2%)	14 (4%)		7 (2%)	14 (4%)	
Stapler side to end	516 (88%)	256 (81%)		272 (86%)	256 (81%)	
Stapler end to end	29 (5%)	22 (7%)		17 (5%)	22 (7%)	
Stapler side to side	31 (5%)	22 (7%)		22 (7%)	22 (7%)	
No anastomosis	0	4 (1%)		0	4 (1%)	
Methods for specimen removal						
Mc Burney’s incision	392 (67%)	125 (40%)	0.026	209 (66%)	125 (40%)	0.038
Pfannenstiel-Kerr incision	136 (23%)	65 (20%)		71 (23%)	65 (20%)	
Not specified	57 (10%)	128 (40%)		38 (12%)	128 (40%)	

In the RSI group, 77% of the operations were indented to be laparoscopically performed, while in the ASI group only 66% were planned by minimally invasive technique (246 vs. 209, *p* = 0.002). In addition, 52 patients underwent robot-assisted (DaVinci Xi®) surgery in the RSI group and 29 in the ASI group (16% vs. 9%, *p* = 0.009). Considering intraoperative conversions from laparoscopic or robotic surgery to open surgery, in ASI 39% of the resections were performed as an open resection whereas 11% of RSI were finished as open surgery (*p* < 0.001).

The technique of anastomosis and operative details are shown in Table 4. All patients in the RSI group received an anastomosis, while four patients (1%) in the ASI group did not receive an anastomosis rather a Hartmann’s procedure was performed (0/318 vs. 4/318, *p* = 0.124). Furthermore, only one patient received a planned defunctioning stoma in the RSI group, whereas there were 27 patients who received a planned defunctioning stoma in the ASI group (0.3% vs. 8.5%, *p* < 0.01) (Table 4).

### Long-term complications

The median duration of follow-up in the RSI group was 75.5 months (IQR 16–125 months) and in the ASI group 68.4 months (IQR 16–125 months). Overall postoperative complications occurred in 44% of patients in the RSI group, while there were 51% of patients in the ASI group, respectively (*p* = 0.068). While there was no difference in minor complications (*p* = 0.689), there were 3.77% and 10% major complications in RSI and ASI, respectively (*p* = 0.003). For further details of major complications refer to Table 5. The mean CCI was 9.4 in the RSI group and 12.9 in the ASI group (*p* = 0.001). There were three anastomotic leaks in RSI group and eight in the ASI group (0.94% vs. 2.52%, *p* = 0.223). Two patients in the RSI group and seven patients in the ASI group received a revisional stoma due to complication (0.6% vs. 2.2%, *p* = 0.177). The overall risk for a stoma formation, including the above mentioned planned defunctioning stomas, was significantly lower in the RSI group than in the ASI group, (3 {0.94%} vs. 38 {10.7%}, *p* < 0.001). In the RSI group, all three stomas were reversed, while in the ASI group 30 of 38 stomas were reversed (3 {100%} vs. 30 {79%}, *p* = 0.001). The mean number of weeks to stoma reversal was 21 weeks in the RSI group and 19 weeks in the ASI group (*p* = 0.769). There was no mortality in either group. The univariate analysis is shown in Table 5.

**Table 5:** Postoperative complications of patients in the RSI and ASI group

	Unmatched (*n* = 903)			Propensity score matching (PSM) (*n* = 636)		
	RSI-G *n* = 585	ASI-G *n* = 318	*p*-value	RSI-G *n* = 318	ASI-G *n* = 318	*p*-value
Follow-up (months, mean, IQR)	73.8 (16–125)	68.4 (16–125)	0.014	75.5 (16–125)	68.4 (16–125)	0.005
Overall postoperative complication rate (*N* = patients, %)	253 (43%)	163 (51%)	0.025	139 (44%)	163 (51%)	0.068
Minor complications (*N*, %)	234 (40%)	139 (44%)	0.29	133 (42%)	139 (44%)	0.689
Clavien-Dindo I	79 (14%)	33 (10%)	0.205	45 (14%)	33 (10%)	0.183
Clavien-Dindo II	133 (23%)	91 (29%)	0.053	76 (24%)	91 (29%)	0.207
Clavien-Dindo IIIa	22 (4%)	15 (5%)	0.487	12 (4%)	15 (5%)	0.695
Major complications, Clavien-Dindo ≥ IIIb (*N*, %)	25 (4.44%)	32 (10%)	0.002	12 (3.77%)	32 (10%)	0.003
Clavien-Dindo IIIb	14 (2.40%)	20 (6.29%)	0.005	6 (1.89%)	20 (6.29%)	0.008
Anastomotic leaks	6 (1.03%)	5 (1.57%)		2 (0.63%)	5 (1.57%)	
Incisional hernias	2 (0.34%)	9 (2.83%)		1 (0.31%)	9 (2.83%)	
Surgical site infection	1 (0.17%)	0		1 (0.31%)	0	
Bleeding	3 (0.51%)	1 (0.31%)		1 (0.31%)	1 (0.31%)	
Anastomositis	1 (0.17%)	0		0	0	
Postoperative acute appendicitis	1 (0.17%)	0		1 (0.31%)	0	
Fistula	0	2 (0.63%)		0	2 (0.63%)	
Stenosis of anastomosis	0	1 (0.31%)		0	1 (0.31%)	
Bowel obstruction	0	1 (0.31%)		0	1 (0.31%)	
Dislocation of Drainage into abdomen	0	1 (0.31%)		0	1 (0.31%)	
Clavien-Dindo IV	11 (1.88%)	12 (3.77%)	0.119	6 (1.89%)	12 (3.77%)	0.231
Anastomotic leaks	1 (0.17%)	2 (0.63%)		1 (0.31%)	2 (0.63%)	
Allergic reaction	1 (0.17%)	0		1 (0.31%)	0	
Pulmonary embolism	1 (0.17%)	2 (0.63%)		0	2 (0.63%)	
Catheter-associated Sepsis	2 (0.34%)	2 (0.63%)		1 (0.31%)	2 (0.63%)	
Bleeding	2 (0.34%)	1 (0.31%)		0	1 (0.31%)	
Clostridium difficile colitis	1 (0.17%)	2 (0.63%)		1 (0.31%)	2 (0.63%)	
Cardiac event	3 (0.51%)	1 (0.31%)		2 (0.63%)	1 (0.31%)	
Renal failure	0	1 (0.31%)		0	1 (0.31%)	
Perforation	0	1 (0.31%)		0	1 (0.31%)	
Mortality (*N*, %)	1 (0.17%)	0	1	0	0	1
Anastomotic leaks	1	0	1	0	0	1
CCI (comprehensive complication index) (mean)	9.3	12.9	< 0.001	9.4	12.9	< 0.001
Anastomotic leaks (*N*, %)	8 (1.37%)	8 (2.52%)	0.290	3 (0.94%)	8 (2.52%)	0.223
Number of stomas (*N*, %)	9 (1.54%)	38 (10.7%)	< 0.001	3 (0.94%)	38 (10.7%)	< 0.001
Hartmann procedure	0	4 (1.3%)	0.152	0	4 (1.3%)	0.152
Planned	1 (0.2%)	27 (8.5%)	< 0.001	1 (0.3%)	27 (8.5%)	< 0.001
Revisional	8 (1.4%)	7 (2.2%)	0.415	2 (0.6%)	7 (2.2%)	0.177
Stoma reversal (*N*, %)	8 (88.89%)	30 (78.95%)	0.001	3 (100%)	30(78.95%)	0.001
Number of weeks to reversal (weeks, mean, IQR)	21.25 (7–36)	18.83 (2–59)	0.608	21 (7–36)	18.83 (2–59)	0.769

## Discussion

Practical and helpful national and international guidelines on diverticular disease exist. They concur and provide clear recommendations regarding emergency surgery and recommend taking patients-related factors into consideration when surgery is up for debate. There also is consensus on elective surgery in patients with chronic complicated diverticulitis with fistula or stenosis [9, 12, 13]; however, there are some nuances amongst guidelines. The German guidelines recommend considering elective surgery in patients with smoldering diverticulitis (ongoing diverticulitis) after one episode of uncomplicated diverticulitis, and surgery should be offered to patients after conservatively or interventionally treated macroabscess (> 3 cm). For patients with recurrent uncomplicated diverticulitis, the quality of life should serve as a decision-making aid for indication for elective surgery [9]. In contrast, the ESCP (European Society of Coloproctolgy) do not differ between uncomplicated or acute complicated diverticulitis. The goal of the elective surgery is to improve quality of life. The indication is individualized based on frequencies of recurrences, duration and frequencies after attack of diverticulitis and co-morbidities of the patients [12]. Similarly, the WSES (World Society of Emergency Surgery) also focuses on the patient-related factors, but additionally, recommends planning elective surgery in immunosuppressed patients, which is not recommended in the German guidelines or by the ESCP [9, 12, 13]. Table 2 shows recommendations regarding elective sigmoidectomy in the most common national and international guidelines from the last decade.

Nevertheless, in the elective setting the indication for surgery still remains vague. The guidelines are more and more reluctant with an indication for sigmoidectomy in recurrent diverticulitis or after a covered perforation. A recent snap-shot analysis across 49 Swiss hospitals showed that 82% of the sigmoidectomies for diverticular disease were performed for recurrent diverticulitis or after covered perforation [20]. According to the current version of the German guidelines [9], there is no indication for elective sigmoidectomy after microabscess (CDD IIa), whereas elective surgery may be offered to patients with a successfully conservatively treated macroabscess (CDD IIb). This is substantiated by the fact that one third of patients after a macroabscess will relapse within one year [9]. However, a prospective observational trial demonstrated that within two years, 60% of patients with an initial microabscess (IIa) and 100% of patients with macroabscess (IIb) needed a sigmoidectomy due to relapse [27].

Furthermore, concerning the indication for surgery in an elective setting, nowadays the quality of life and the risk of complications should be included. It is well established that quality of life can be reduced after diverticulitis compared to healthy people [28]. 36% of the patients have gastrointestinal complaints after conservative treatment of diverticulitis [29] and the randomized DIABOLO study showed as well, that one third of the patients had a relevant reduction in quality of life, regardless of whether they had been treated or supervised with antibiotics [30]. In addition, 4–10% of patients develop smoldering diverticulitis despite antibiotic therapy [31, 32] and recurrences rates after drug-treated diverticulitis were reported between 13 and 36%, depending on the population studied and the duration of the treatment [33]. The need for emergency surgery after acute diverticulitis is 2–14% and the risk of stoma creation or death of 0–2.7% over a 5-year follow-up period [34]. For these reasons an elective sigmoid resection in symptomatic recurrent diverticulitis might be decided after a balanced risk–benefit analysis.

A risk assessment for postoperative complications after elective sigmoidectomy in relation to patient-related factors and severity of disease does not exist. In this study, peri- and postoperative surgical outcomes in two homogenous groups after PSM of patients with different severity of indication requiring elective sigmoidectomy were compared. The study demonstrated that the risk profile in these two groups differ largely. Patients that underwent sigmoidectomy for recurrent diverticulitis or after microabscess (RSI) had an 11-fold decreased risk for the need of a defunctioning stoma, a threefold decreased risk for open surgery and a 2.6-fold decreased risk for major morbidity. In chronic complicated diverticulitis (ASI), the proportion of minimally invasive surgery was smaller and the intraoperative conversion rate to open surgery was higher (14% in ASI versus 5% in RSI). Other studies have reported conversion rates of between 2.3% and 23%, but with lack of consistent significant difference between complicated and uncomplicated diverticulitis [35–37].

The operation time was ten minutes longer in the ASI group compared to the RSI group. Although the difference is significant, its clinical relevance is negligible. Mizrahi et al. provided outcomes consistent with our results regarding surgery time, comparing patients with complicated versus uncomplicated diverticulitis (218 min. vs. 185 min.). However, no open procedures were performed in this study [21]. Furthermore, Williams et al. investigated surgery duration in elective laparoscopic sigmoidectomy and showed a significant association between operation time and older age, greater BMI, concurrent hypertension and higher ASA classification, as well as the presence of dense adhesions intraoperatively, fistulas and abscesses. However, the data do not support pre-emptive conversion of laparoscopic sigmoidectomy to avoid prolonged operative times [22]. Our study shows similar association due to the presence of more severe diverticular disease with complicated diverticulitis in the ASI group (CDD Type IIb and IIIc). It is quite understandable that with more severe courses of disease, more difficult intraoperative situations are entailed. Nevertheless, our data also supports the laparoscopic approach if progress is safely being made, especially since intraoperative anesthesiological problems or complications are rare (0.3% in the ASI group).

In the two groups, a comparably low incidence of intraoperative complications was observed (3% vs. 5% in RSI vs ASI, respectively), the most frequent being intraoperative bleeding in different locations (0.6% vs. 3.1% in RSI vs ASI, respectively). This might also be due to difficult scarring conditions in more severe disease courses in the ASI group. Other intraoperative complications were comparable to those reported in other studies for elective sigmoidectomy for diverticular disease [24, 25, 37].

This study showed an overall complications rate of 44% in the RSI group and 51% in the ASI group (*p* = 0.068). Similar rates were found in minor complications (Clavien-Dindo < IIIb) with 42% in the RSI group and 44% in the ASI group (*p* = 0.689). Important to note, the analysis on postoperative complications in this study was performed very accurate and any documented deviation from normal course has been included in this study for analysis, and most of the complications were Clavien-Dindo I and II (38% in RSI vs. 39% in ASI). Therefore, in our point of view, this is the reason why the complications rate in this study is higher when compared to other studies [10, 11, 21, 24, 25, 35, 36, 38]. RSI patients showed a lower CCI compared with patients in the ASI group (9.4 vs. 12.9). Further data regarding the CCI in patients following elective sigmoidectomy for diverticulitis are currently non-existent.

Intriguingly, the results of postoperative major complications showed a difference between the analysed groups. In the RSI group, postoperative major complication occurred in 3.8% while 10% occurred in the ASI group. Most of the surgery-related complications were anastomotic leaks and incisional hernias. Mizrahi et al. provided a major complication rate of 1% in patients with uncomplicated diverticulitis and 3% in patients with complicated diverticulitis. However, it should be noted again that open surgery had been excluded [21]. Furthermore, the largest case series by Jones et al. that included 500 laparoscopic resections, showed a postoperative major complication rate similar to this study of 11.5% in patients with complicated diverticulitis and 10.9% in patient with uncomplicated diverticulitis respectively, but without a statistically significant difference [35]. On the other hand, Martel et al. reported that patients with severe disease experienced a significantly higher rate of surgical complications and general medical complications [37].

In the present study, 10.7% of ASI patients and 0.9% of RSI patients left the hospital with a stoma. In accordance with these findings, Martel et al. recorded a higher rate of defunctioning ileostomy in complicated versus uncomplicated disease (4.2% vs. 21%, respectively, *p* < 0.05) [37]. Even higher rates of ileostomy creation were reported by Royds et al. (5.2% vs. 35%, *p* < 0.05) [36]. These results show an association with the progression of the disease and creation of ileostomy. One reason for this high difference is not in itself very likely the progression of the disease, but the experience of the surgeon, who associates a previous more severe course of disease with a higher rate of anastomosis leak. Furthermore, chronic colonic ileus in sigmoid stenosis and chronic inflammation due to macroabscess may increase the estimated risk for anastomotic insufficiency. In our studies, surgery was performed or assisted by highly experienced colorectal surgeons, both in the RSI group and in the ASI group. Although creation of ileostomy is not considered a complication, it carries a considerable burden for the patients and may imply potential future complications to the stoma itself or to future surgery.

The retrospective design is accompanied with an inherent risk of selection bias. Neither the follow-up, nor the hospital stay and quality of life were included in the data collected. One strength is the analysis, including most influential co-morbidities and the meticulous collection of data performed with the four-eyes principle. However, history of tobacco smoking, which is also known as a risk factor for complicated diverticulitis, was not recorded. Elective sigmoidectomy were performed or assisted by six highly experienced colorectal surgeons. Although institutional standard operating procedures (SOP) were strictly followed, potential operator bias due to possible differences between surgeons for treatment and surgical technique may exist.

When performing adequate benefit-risk assessment with every individual patient, it is of utmost importance to inquire about quality of life. Several studies investigating quality of life in patients with recurring or ongoing diverticulitis lend themselves to support the benefits of surgery. The DIRECT trial resulted in a significantly increase quality of life at five-year follow-up compared with conservative management and therefore supports surgery despite inherent risk of complications of operative management [10]. Furthermore, the LASER trial comparing laparoscopic elective sigmoid resection with conservative treatment in a randomized setting showed a significant improvement of quality of life after surgery with a 10% risk of major complications [11].

## Conclusion

The current study demonstrates that the surgical risk in terms of major complications, complications burden (CCI), risk of receiving a defunctioning stoma and the risk for the need of open surgery is distinctly different between recurring uncomplicated diverticulitis or after microabscess compared to more advanced disease stages such as after a macroabscess or in case of chronic complicated diverticulitis. The findings of this study will allow more tailored counseling when it comes to the shared decision making between patient and surgeon for or against elective sigmoidectomy.

## Data Availability

The data that support the findings of this study are available from the corresponding author upon reasonable request.

## References

[1] Köhler L, Sauerland S, Neugebauer E (1999). Diagnosis and treatment of diverticular disease: Results of a consensus development conference. Surg Endosc.

[2] Ambrosetti P, Robert JH, Witzig JA (1994). Acute left colonic diverticulitis in young patients. J Am Coll Surg.

[3] Rose J, Parina RP, Faiz O (2015). Long-term outcomes after initial presentation of diverticulitis. Ann Surg.

[4] Ritz J-P, Lehmann KS, Frericks B (2011). Outcome of patients with acute sigmoid diverticulitis: multivariate analysis of risk factors for free perforation. Surgery.

[5] Boermeester MA, Humes DJ, Velmahos GC, Søreide K (2016). Contemporary review of risk-stratified management in acute uncomplicated and complicated diverticulitis. World J Surg.

[6] Pittet O, Kotzampassakis N, Schmidt S (2009). Recurrent left colonic diverticulitis episodes: more severe than the initial diverticulitis?. World J Surg.

[7] Leifeld L, Germer CT, Böhm S (2014). S2k guidelines diverticular disease/diverticulitis. Z Gastroenterol.

[8] Horesh N, Wasserberg N, Zbar AP (2016). Changing paradigms in the management of diverticulitis. Int J Surg.

[9] Leifeld L, Germer CT, Böhm S (2022). S3-Leitlinie divertikelkrankheit/divertikulitis-gemeinsame leitlinie der deutschen gesellschaft für gastroenterologie, verdauungs-und stoffwechselkrankheiten (DGVS) und der deutschen gesellschaft für allgemein-und viszeralchirurgie (DGAV). Z Gastroenterol.

[10] van de Wall BJM, Stam MAW, Draaisma WA (2017). Surgery versus conservative management for recurrent and ongoing left-sided diverticulitis (DIRECT trial): an open-label, multicentre, randomised controlled trial. Lancet Gastroenterol Hepatol.

[11] Santos A, Mentula P, Pinta T (2021). Comparing laparoscopic elective sigmoid resection with conservative treatment in improving quality of life of patients with diverticulitis: the laparoscopic elective sigmoid resection following diverticulitis (LASER) randomized clinical trial. JAMA Surg.

[12] Schultz JK, Azhar N, Binda GA (2020). European Society of Coloproctology: guidelines for the management of diverticular disease of the colon. Colorectal Dis.

[13] Sartelli M (2020). 2020 update of the WSES guidelines for the management of acute colonic diverticulitis in the emergeny setting. World J Emerg Surg.

[14] Galetin T, Galetin A, Vestweber K-H, Rink AD (2018). Systematic review and comparison of national and international guidelines on diverticular disease. Int J Colorectal Dis.

[15] Sartelli M, Viale P, Catena F (2013). 2013 WSES guidelines for management of intra-abdominal infections. World J Emerg Surg.

[16] Sartelli M, Catena F, Ansaloni L (2016). WSES Guidelines for the management of acute left sided colonic diverticulitis in the emergency setting. World J Emerg Surg.

[17] Andeweg CS, Mulder IM, Felt-Bersma RJF (2013). Guidelines of diagnostics and treatment of acute left-sided colonic diverticulitis. Dig Surg.

[18] Binda GA, Cuomo R, Laghi A (2015). Practice parameters for the treatment of colonic diverticular disease: Italian Society of Colon and Rectal Surgery (SICCR) guidelines. Tech Coloproctol.

[19] Slankamenac K, Graf R, Barkun J (2013). The comprehensive complication index: a novel continuous scale to measure surgical morbidity. Ann Surg.

[20] Faes S, Hübner M, Demartines N (2021). Elective surgery for diverticulitis in Swiss hospitals. Front Surg.

[21] Mizrahi I, Abu-Gazala M, Fernandez LM (2021). Elective minimally invasive surgery for sigmoid diverticulitis: operative outcomes of patients with complicated versus uncomplicated disease. Colorectal Dis.

[22] Williams J, Stocchi L, Aiello A (2021). No need to watch the clock: persistence during laparoscopic sigmoidectomy for diverticular disease. Surg Endosc.

[23] R Core Team (2021) R: A language and environment for statistical computing. R Foundation for Statistical Computing, Vienna. https://www.R-project.org

[24] Raskin ER, Keller DS, Gorrepati ML, et al (2019) Propensity-matched analysis of sigmoidectomies for diverticular disease. JSLS 23. 10.4293/JSLS.2018.00073 10.4293/JSLS.2018.00073 PMC6328361 30675092

[25] Bouillot JL, Berthou JC, Champault G (2002). Elective laparoscopic colonic resection for diverticular disease: results of a multicenter study in 179 patients. Surg Endosc.

[26] Dindo D, Demartines N, Clavien P-A (2004). Classification of surgical complications: a new proposal with evaluation in a cohort of 6336 patients and results of a survey. Ann Surg.

[27] Lauscher JC, Lock JF, Aschenbrenner K (2021). Validation of the German classification of diverticular disease (VADIS)-a prospective bicentric observational study. Int J Colorectal Dis.

[28] Bolster LT, Papagrigoriadis S (2003). Diverticular disease has an impact on quality of life - Results of a preliminary study. Colorectal Dis.

[29] Andeweg CS, Berg R, Staal JB (2016). Patient-reported outcomes after conservative or surgical management of recurrent and chronic complaints of diverticulitis: systematic review and meta-analysis. Clin Gastroenterol Hepatol.

[30] Van Dijk ST, Daniels L, De Korte N (2019). Quality of life and persistent symptoms after uncomplicated acute diverticulitis. Dis Colon Rectum.

[31] Boostrom SY, Wolff BG, Cima RR (2012). Uncomplicated diverticulitis, more complicated than we thought. J Gastrointest Surg.

[32] Daniels L, Ünlü, de Korte N,  (2017). Randomized clinical trial of observational versus antibiotic treatment for a first episode of CT-proven uncomplicated acute diverticulitis. Br J Surg.

[33] Humes DJ, Spiller RC (2014). Review article: The pathogenesis and management of acute colonic diverticulitis. Aliment Pharmacol Ther.

[34] Binda GA, Amato A, Serventi A, Arezzo A (2012). Clinical presentation and risks. Dig Dis.

[35] Jones OM, Stevenson ARL, Clark D (2008). Laparoscopic resection for diverticular disease: follow-up of 500 consecutive patients. Ann Surg.

[36] Royds J, O’Riordan JM, Eguare E (2012). Laparoscopic surgery for complicated diverticular disease: a single-centre experience. Colorectal Dis.

[37] Martel G, Bouchard A, Soto CM (2010). Laparoscopic colectomy for complex diverticular disease: a justifiable choice?. Surg Endosc.

[38] Ilyas MIM, Zangbar B, Nfonsam VN (2017). Are there differences in outcome after elective sigmoidectomy for diverticular disease and for cancer? A national inpatient study. Colorectal Dis.

